# Should Mini-Thoracotomy Be the Preferred Anterior Approach to the Thoracic Spine?

**DOI:** 10.7759/cureus.110379

**Published:** 2026-06-07

**Authors:** Yan Ting Woo, Shreya Saraf, Zaeem Sultan, Marcin Czyz

**Affiliations:** 1 Medical School, University of Birmingham College of Medical and Dental Sciences, Birmingham, GBR; 2 Neurosurgery, University Hospitals Birmingham NHS Foundation Trust, Birmingham, GBR

**Keywords:** anterior approach, disc herniation, minimally-invasive spine, spinal malignancy, thoracic sugery, thoracotomy

## Abstract

Objectives

To compare the short- and mid-term surgical outcomes in patients undergoing mini-thoracotomy (MT) and conventional open thoracotomy (COT) as anterior approaches to the thoracic spine.

Methods

Data were collected for adults who underwent thoracic spine procedures via an anterior surgical approach for various indications at the Department of Neurosurgery, Queen Elizabeth Hospital Birmingham (QEHB), between 2016 and 2021. Electronic medical records and clinical imaging of the patients enrolled on the study were reviewed. Primary outcomes measured were complications and post-operative pain levels. Secondary outcomes included the lengths of hospitalisation and intensive care unit (ICU) stays, estimated blood loss (EBL), duration and output of the post-operative chest drain, operation time, and radiation exposure. These outcomes were then compared between the MT and COT cohorts. The software, IBM SPSS Statistics, version 28 (IBM Corp., Armonk, NY, USA), alongside parametric and non-parametric tests, was used, with a p-value set at <0.05.

Results

A total of 31 patients (18 females and 13 males) with an average age of 53±15 were included. Fourteen underwent MT, while 17 had COT. There were no significant demographic differences between the cohorts. MT resulted in fewer complications, but the difference was not statistically significant. Pain levels were lower at 48 hours post-operation but higher on discharge in MT. Secondary outcome results yielded that the MT group had a statistically significant 80% shorter post-operative ICU stay than the COT group (p=0.034). Duration of the procedure and EBL were comparable in both groups. The differences between duration and output of the chest drain and post-operative opioid use favoured MT, but were not statistically significant.

Conclusion

Based on our study, MT is non-inferior to COT. MT seemed related to shorter post-operative ICU stay and is potentially positively associated with the other outcomes. However, studies involving larger patient cohorts are required to verify the statistical significance and clinical relevance of these observations.

## Introduction

Thoracic spinal lesions such as fractured vertebrae, slipped discs, and oncological masses are often approached anteriorly as this allows for direct access to lesions [1]. The conventional open thoracotomy (COT) remains the gold standard procedure for this approach [2]. However, there is increasing evidence that mini-thoracotomy (MT), a less invasive surgical procedure, may be an emerging alternative to the conventional open approach [2,3]. Although the current evidence base assessing the value of MT in a neurosurgical setting is limited, it suggests that MT may be a superior approach to COT by way of smaller incisions, less tissue damage, and fewer incidences of complications [4-6]. The minimally invasive approach also results in an improved cosmesis, earlier mobilisation, and faster recovery [6].

Notably, the majority of research into the benefit of MT has been done in cardiothoracic context [3,7,8]. This gives rise to the question: are these benefits also seen in neurosurgical patients? This paper therefore aims to evaluate the effectiveness of MT as compared to COT for neurosurgical patients, in order to maximise patient benefit and ensure best practice. Our hypothesis is that MT is at least a non-inferior approach when compared to COT for selective patients.

## Materials and methods

This cohort study was carried out retrospectively. Data were procured for all anterior thoracotomy procedures performed on adults at the Department of Neurosurgery at the Queen Elizabeth Hospital Birmingham (QEHB), from 2016 to 2021. The databases used for the search were PICS (Prescribing Information Communication System), PACS (Picture Archiving and Communication Systems) and Clinical Portal, which are databases utilised in the National Health Service (NHS). Cases adopting other approaches (such as lateral and posterior approaches) to the thoracic spine were excluded. This yielded a total of 31 cases for analysis. For the purpose of our study, we define MT as surgical approaches with small incision sizes (5-6 cm as compared to approximately 30 cm in COT), non-removal of ribs, as well as reduced dissection of intercostal muscles, as compared to COT. Cases with the MT approach were then identified from these 31 cases.

For analysis, we looked at several variables, which were grouped into primary and secondary outcomes. The primary outcomes were complication rates and post-operative pain levels (both at 48 hours post-operation and on discharge) measured through the Visual Analogue Scale and opioid usage. Secondary outcomes were lengths of hospitalisation and intensive care units (ICU) stays, estimated blood loss (EBL; as approximated through the difference between pre- and post-operative haemoglobin levels), duration and output of chest drain, operation time, and radiation exposure. Results of these outcomes were then compared between MT and COT, and subsequently analysed to determine whether patients who underwent MT had better patient outcomes.

In terms of data analysis, we used the software IBM SPSS Statistics, version 28 (IBM Corp., Armonk, NY, USA). The Shapiro-Wilk test was first conducted to determine the normality of each variable. For those with a normal distribution, an independent-sample T test was adopted to determine the statistical significance. The Mann-Whitney U test was used for the remaining variables.

## Results

In total, we had 14 cases of MT and 17 cases of COT in our study (Table 1).

**Table 1:** Demographics of included patients

Charactersistics	Mini-thoracotomy	Conventional open thoracotomy
Total number of patients	14	17
Gender, N (%)		
Male	6 (43)	7 (41)
Female	8 (57)	10 (59)
Age, Mean +/- SD	53.64 +/- 15.55	52.65 +/- 14.55

**Figure 1:**
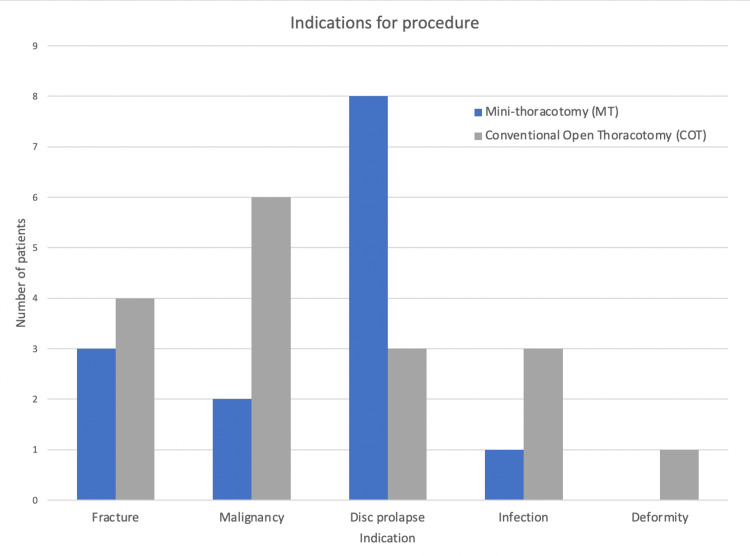
Types of indications for mini-thoracotomy (MT) and conventional open thoracotomy (COT) with the respective distributions

Upon analysis of the demographics of the two groups of patients, we found that the age and gender of patients were not statistically different between the two groups. We categorised the indications for both procedures into five main types, as shown in Figure 1. It was noted that the type of approach appeared to vary depending on the indication of surgery, with disc prolapse forming the bulk of indications to perform MT, whereas malignancy was the most common reason to carry out COT. The only indication which was more equally distributed between the two groups was spinal fracture, with 3/7 patients (43%) having undergone MT.

Table 2 summarises the primary outcome findings. We noted that overall, COT had a higher frequency of complications, with complications seen in 13/17 (76%) of the COT cases as compared to 4/14 (29%) of the MT cases.

**Table 2:** Summary of the results under the primary outcomes with the associated conclusions derived *Median values comparing all outcomes; ┼On the pain Visual Analogue Scale; ▲In milligrams of oral morphine

Primary outcomes*		Mini-thoracotomy (n=14)	Conventional open thoracotomy (n=17)	Conclusion	Independent Sample T-test/Mann-Whitney U value	P-value
Complication frequency		29%	76%	Mini approach resulted in fewer complications	U = 97.0	0.399
Reported Pain Score^┼^	48h post-operation	0.5	1	Mini approach had lower pain scores reported in the immediate post-operative period	U = 105.5	0.790
	On discharge	1.3	0	Conventional approach had lower pain scores reported on discharge	U = 93.5	0.316
Opioid use^▲^	48h post-operation	50 mg	60 mg	Mini approach had 17% less opioid dosage in the immediate post-operative period	U = 116.5	0.922
	On discharge	16 mg	20 mg	Mini approach had 20% less opioid dosage on discharge	U = 115.0	0.891

**Figure 2:**
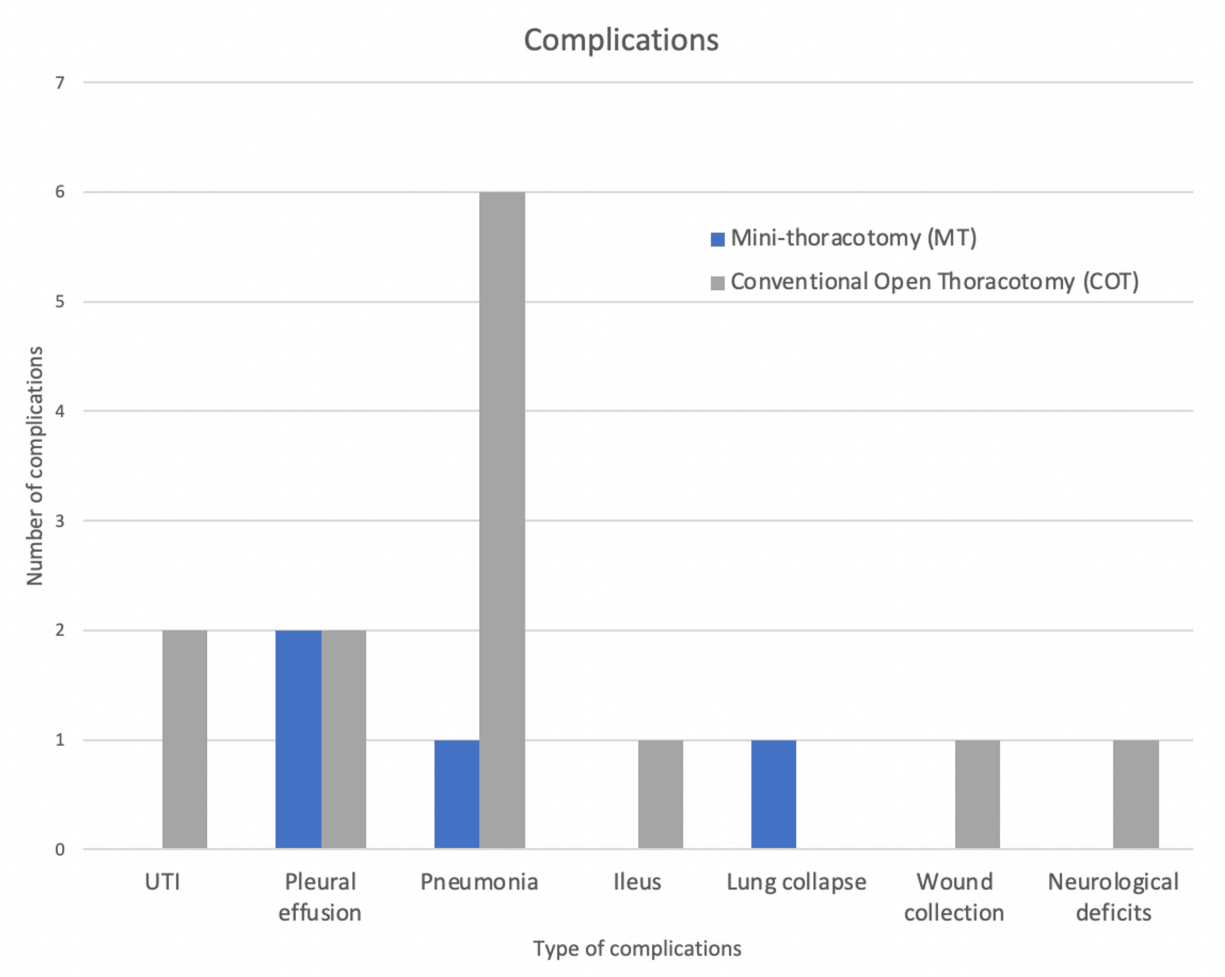
Types of complications and the respective distributions between mini-thoracotomy (MT) and conventional open thoracotomy (COT)

In terms of the types of complications that occurred, there were mainly seven types seen in both groups (Figure 2). The most common complication in general was pneumonia, which formed 7/17 (41%) of all complications. Of these, 6/7 (86%) were seen in COT. This is followed by pleural effusion being the second most common complication (4/17 cases, 24%), with an equal spread (two cases each) in both approaches.

Pain levels were measured using VAS scores and opioid usage. For both groups, VAS pain scores reported by patients both at 48 hours post-operation and on discharge were collected. MT had a lower reported pain score at 48 hours post-operation, with a median of 0.5 as compared to 1.0 in the COT cohort (p-value = 0.790). On discharge, COT had a lower median pain score of 0, as opposed to 1.3 for MT (p-value = 0.316). Similarly, we collected data on the amount of opioids used both at 48 hours post-operation and on discharge, using a standard of oral morphine in milligrams to compare. MT had resulted in a smaller amount of opioids being used at 48 hours post-operation, with a median of 50 mg as compared to 60 mg in COT (p-value = 0.922). On discharge, a median of 16 mg of opioids was used in MT, whilst a median of 20 mg of opioids was utilised in COT (p-value = 0.891).

Secondary outcomes measured are summarised in Table 3. A statistically significant difference was noted in the length of ICU admission, with patients undergoing COT remaining on ICU for five times as long as those undergoing MT (p-value = 0.034). Despite the lack of statistical significance, MT was also seen to result in more beneficial results in other parameters. MT had resulted in a shorter hospital stay of 12 days than 17 days of COT (p-value = 0.149). Operation time was longer by just under eight minutes in the COT group (3.25 hours in MT vs 3.38 hours in COT, p-value = 0.608). Both chest drain duration and output were lower in MT - two days and 210 millilitres, respectively, as compared to two and a half days (p-value = 0.288) and 410 millilitres (p-value = 0.183) in COT. Radiation exposure, however, favoured patients undergoing COT, with this cohort experiencing half as much radiation as the MT group (p-value = 0.131). Estimated blood loss was also slightly less in COT (Hb drop of 11 vs 14 in MT, p-value = 0.442).

**Table 3:** Summary of the results under the secondary outcomes and conclusions drawn *Median or mean values comparing all outcomes; ┼Estimated using 48h pre- and post-operative haemoglobin levels

Secondary outcomes*		Mini-thoracotomy (n=14)	Conventional open thoracotomy (n=17)	Conclusion	Independent Sample T-test/Mann-Whitney U value	P-value
Length of admission (days)	Total duration	12	17	Mini approach resulted in 29% shorter hospital admission	U = 82.0	0.149
	ICU duration	2.5	5	Mini approach resulted in 80% shorter ICU admission	t = -2.23	0.034
Estimated blood loss (Hb difference in g/dL)^┼^		-18.5	-12.4	Drop in haemoglobin was slightly less in conventional approach	t = -0.79	0.442
Chest drain	Duration (days)	2	2.5	Mini approach resulted in 20% shorter duration of chest drain placement	U = 79.5	0.288
	Output (mLs)	210	410	Mini approach resulted in 49% lower chest drain volume output	U = 62.0	0.183
Estimated operative time (hours)		3.24	3.59	Duration of procedure was shorter in mini approach	t = -0.52	0.608
Radiation exposure (mGym^2^)		0.32	0.14	Conventional approach had 52% less radiation exposure	t = 1.66	0.131

## Discussion

Open thoracotomy is the conventional anterior surgical approach, in which an incision is made in the intercostal space to gain surgical access to the thoracic spine. On the other hand, MT is a type of minimally invasive surgical method which has gained popularity over the years due to the associated benefits over COT [4-6]. The main difference between the two includes, as the name implies in MT, a significantly shorter skin incision and less extensive dissection of the intercostal muscles. The rib at the level of the approach remains intact or is divided proximally and then reconstructed during the closure in MT.

Based on our study outcome, MT is potentially associated with several benefits as compared to COT, but due to the statistical insignificance of the results, larger studies will be required to better establish the correlations. However, in our study, MT is at least non-inferior to COT and should therefore continue to be performed.

As mentioned earlier, MT seemed to have potentially resulted in better outcomes than COT, including fewer complications, less post-operative pain, reduced opioid usage post-operation and on discharge, shorter hospital admission and ICU stay, shorter duration of the chest drainage with lower overall output and shorter operation time. On the other hand, COT was related to lower pain levels on discharge (perhaps owing to higher opioid usage in this group), lower blood loss and less radiation exposure. However, it is important to note that only the ICU stay was shown to be a statistically significant difference. The discussions below regarding the other results would not be conclusive. Further studies are necessary for more definitive evidence.

The most notable statistically significant secondary outcome measured in our study was the length of ICU admission, which was five times less in patients undergoing MT than its conventional counterpart. Reduced ICU admission duration is a vital finding for patients’ physical health and mental well-being, as suggested by evidence analysing morbidity and mortality in relation to ICU stay [9,10].

Despite not being statistically significant, the difference in pain experienced by patients who had undergone MT and COT, respectively, both immediately post-operation and on discharge, should be looked into. It is essential to minimise pain experienced by patients undergoing thoracotomy procedures, so as to enhance their recovery through the various benefits brought about by analgesia optimisation [11,12]. These pain scores should be analysed in line with the analgesia used by the patients. The reasons for the reverse trend in pain experienced by patients in our study (i.e., MT resulting in lower pain scores post-operatively but higher pain scores on discharge) are unclear. The use of anticipatory analgesia could be a potential contributing factor, but it does not fully explain the post-operative pain, as those with MT had less pain but also less analgesics used. Again, further research would be needed to identify the patterns in pain experienced from the procedures and analgesia usage, due to the impact on patient recovery as mentioned above, as well as the potential harmful effects related to opioid use as a form of analgesia [13,14]. 

With regards to the lower frequency of complication occurrence in MT, it could be attributed to several factors, including smaller incision sizes and a less invasive approach requiring less support with a shorter ICU stay, as illustrated by our results.

The doubled radiation exposure in patients from the MT group can likely be accounted for by the nature of the procedure; the narrow incision and precise placement of retractors (if used) likely required more frequent imaging than needed in conventional procedures with larger incisions. Operation time was less in the MT group by around eight minutes; however, these procedures were performed by only a handful of surgeons with experience and expertise in doing them. It is likely that during the initial learning curve of implementing this procedure, MT may take longer to do as surgeons build dexterity. It was shown that, depending on the complexity of the surgery, there might be a certain number of cases that surgeons tend to reach before achieving their best performance [15]. It is anticipated that once this is achieved, the procedure will take a shorter duration of time, and this time efficiency is of great value, particularly in a public health setting like the National Health Service in the UK.

Despite attempts to minimise the limitations in our study, there are several main factors which could potentially affect the reliability of our study results - small sample size, unclear baseline status of the patients and heterogeneity of diagnoses - which are some of the expected limitations of a retrospective study design. As the MT approach is a relatively new procedure, the sample size of our study remains small, making it a possible limitation in terms of data transferability. The small sample size has also likely contributed to most of the results being non-statistically significant in our study. Although there was no significant demographic difference between the two patient groups, pre-operative performance status scores were not recorded for most patients. Unfortunately, there was insufficient information recorded in the notes to score the patients retrospectively, and it is possible that the performance status of patients affected which approach they were suited to having, and possibly influenced their recovery outcomes. Furthermore, the heterogeneity of diagnoses in both groups, with disc prolapse being the most common indication for MT and malignancy for COT, might imply that accurate comparison between the two groups would be difficult. Any disease spread in malignancy might also favour a wider exploratory approach that the COT offers over MT. This heterogeneity might have also contributed to the data obtained; for example, patients with malignancies might be more likely to experience pain post-operatively than those suffering from disc prolapse.

Apart from these, we noted that blood loss was not consistently documented in the post-operative notes. We therefore estimated by comparing the most recent pre- and post-operative haemoglobin levels available for each patient. Although this was not the most precise means of determining blood loss, it was the most accurate method possible with the available data.

To overcome these shortcomings, an extended subgroup analysis would be beneficial, such as one based on the different diagnoses, as well as more precise documentation in future surgical assessments to include factors such as EBL and performance status.

Despite the limitations, our study demonstrated that MT is at least non-inferior to COT. This study could therefore serve as a starting point for further research in MT. Our data has demonstrated the shorter ICU stay associated with MT, which was statistically significant. Future studies analysing the impact of MT would be necessary to acquire more definitive answers regarding the other parameters.

MT could be a promising anterior surgical approach to thoracic spinal lesions. Having said this, it is important to consider the cost-benefit implications of a newer surgical approach, which might affect the decision-making of practising MT as a first-line approach in suitable patients. Further research into the cost-benefit analysis of MT would be useful, as elaborated above.

## Conclusions

MT is likely non-inferior to COT. Based on our study, MT was associated with a shorter post-operative ICU stay as compared to COT, as well as potentially better outcomes in post-operative pain, opioid use both post-operatively and at discharge, chest drain duration and output, hospital and ICU lengths of stay, and operative time. These factors are important when considering the most suitable surgical approach for patients, as ICU admission, pain management and other peri-operative factors may influence post-operative recovery. Based on our findings, MT represents a viable anterior approach to thoracic spinal lesions and appears to be non-inferior to COT. However, further studies involving larger patient cohorts are required to confirm the statistical significance and clinical relevance of these observations, and to help inform clinical practice.
